# Measurement and Modeling of Semi-Batch Solution Radical Copolymerization of *N-tert*-Butyl Acrylamide with Methyl Acrylate in Ethanol/Water

**DOI:** 10.3390/polym15010215

**Published:** 2022-12-31

**Authors:** Gagandeep Kaur, Maryam Agboluaje, Robin A. Hutchinson

**Affiliations:** Department of Chemical Engineering, Queen’s University, 19 Division St., Kingston ON K7L 3N6, Canada

**Keywords:** radical polymerization, polymerization kinetics, copolymerization, reactivity ratios, semi-batch reaction, methyl acrylate, *N*-*tert*-butylacrylamide, hydrogen bonding, solvent effects

## Abstract

The synthetic polymer industry is transitioning from the use of organic solvents to aqueous media in order to reduce environmental impact. However, with radical polymerization kinetics affected by hydrogen-bonding solvents, there is limited information regarding the use of water as a solvent for sparingly soluble monomers. Thus, in this paper, the radical polymerization of methyl acrylate (MA) and *N*-*tert*-butylacrylamide (t-BuAAm) is studied in water and ethanol (EtOH), as the copolymer product is of commercial interest. A series of semi-batch reactions are conducted under a range of operating conditions (i.e., reaction temperature, solvent-to-monomer ratio, and comonomer composition) to demonstrate that the copolymer can be successfully synthesized without significant drifts in product molar masses or composition. The experiments provide additional data to probe the influence of the solvent on the polymerization rate and copolymer properties, as the low monomer concentration maintained under starved-feed operation leads to a solvent-to-monomer ratio different from that in a batch system. A model that captures the influence of backbiting and solvent effects on rate, previously developed and tested against batch polymerizations, also provides an excellent description of semi-batch operation, validating the set of mechanisms and kinetic coefficients developed to represent the system.

## 1. Introduction

Free radical polymerization has been widely used to produce a diverse range of commercial products for decades [1]. With advancements in technology, there is a continuous push to create modified products with consistent characteristics. Acrylamide-acrylate copolymer solutions are replacing more complex mixtures used in consumer personal care items, including in hair gels, creams, and waxes [2]. Solvent is an essential part of commercial recipes, as it reduces viscosity and improves heat removal from the reaction systems designed to produce copolymers with uniform properties. While many commercial systems use non-polar organic solvents for synthesis, these are being replaced by eco-friendly and biocompatible solvents, such as water and alcohols, particularly for biomedical and personal care product applications [3,4]. However, polar solvents can form hydrogen bonds (H-bonds) with monomers and/or disrupt monomer–monomer H-bonding, affecting polymerization kinetics and polymer properties compared to the bulk system or polymerization in a non-polar solvent. With few literature studies of solution radical polymerization kinetics of partially water-miscible monomers available, this work studies the polymerization of methyl acrylate (MA) and *N*-*tert*-butylacrylamide (t-BuAAm) in a solution mixture of ethanol (EtOH) and H_2_O. 

Agboluaje et al. [5] applied the pulsed-laser polymerization–size-exclusion chromatography (PLP-SEC) technique to study the effect of an alcohol/water solvent on the propagation rate coefficient ($k_{p}$) of both MA and a second sparingly soluble monomer, 2-methoxyethyl acrylate (MEA). The $k_{p}$ values increased both with a decreasing monomer concentration in the solution and with an increasing fraction of water in the alcohol/water mixtures: at 30 °C, the $k_{p}$ for 5 wt% MA (*w_mon_* = 0.05) in an EtOH/H_2_O mixture containing a weight fraction of ethanol ($\alpha_{\mathrm{EtOH}}$) of 0.75 was 3 times greater than the bulk value, with the value increasing to a factor of 16 higher in pure water [5]. The experimental data for MA were well-represented by the following:

(1) $$k_{p}^{MA}\left(\frac{L}{\mathrm{mol}.s}\right)=k_{p,bulk}^{MA}\left[A+\left(1-A\right)\exp\left(-C.{w^{\prime }}_{sol}\right)\right]$$

$${w^{\prime }}_{sol}=1-{w^{\prime }}_{mon};\;A=0,\;\;\;C=a+b$$

$$a=\left[-0.0075{\left(\alpha_{EtOH}-0.4753\right)}^{2}+0.0164\right]\left(T-T_{ref}\right)$$

$$b=-0.8398{\left(\alpha_{EtOH}-1.7471\right)}^{2}-0.3644$$

$$k_{p,bulk}^{MA}\left(\frac{L}{\mathrm{mol}.s}\right)=1.41\times 10^{7}\exp\left(-\frac{17,300}{RT}\right)$$

where *T* is the temperature in kelvin, the reference temperature *T_ref_* is 303 K, *R* is the universal gas constant in J/mol·K, the weight fraction of the solvent ${w^{\prime }}_{sol}$ is calculated on a polymer-free basis, and the Arrhenius equation for $k_{p,bulk}^{MA}$ is taken from an IUPAC benchmark study [6]. 

Previous studies have shown that the polymerization of acrylamides is also affected by the reaction medium. Ganachaud et al. reported that the $k_{p}$ of *N*-isopropyl acrylamide increases by a factor of 2 at infinite dilution compared to ${w^{\prime }}_{sol}$ = 0.95 in water [4]. Solvent composition also plays a role, with the overall rate of acrylamide polymerization the highest in water and decreasing with the addition of alcohol to the solvent mixture [7]. Recently, the PLP-SEC technique has been applied to determine that the $k_{p}$ value for t-BuAAm, a monomer with reduced water solubility compared to MA, increases by 15% when $\alpha_{\mathrm{EtOH}}$ is reduced from 1 to 0.75 for *w_mon_* = 0.10 at 30 °C [8]. 

In addition to its influence on chain-growth kinetics in polar solutions, the monomer concentration also impacts the relative importance of backbiting on the polymerization rate in acrylate and acrylamide systems. The intramolecular H-atom abstraction process (i.e., backbiting, with rate coefficient $k_{bb})$ transfers the radical functionality from the chain-end secondary propagation radical (SPR) to a tertiary carbon-centered mid-chain radical (MCR). As the configuration of the MCR is more stable than that of the SPR due to its tertiary nature, the propagation rate coefficient for the monomer addition to the MCR ($k_{p,tert}$) is much less than that for the addition to the SPR $\left(k_{p}\right)$, resulting in a reduction in the rate of overall monomer consumption. The influence can be captured by defining an effective propagation rate coefficient ($k_{p,eff})$ that is lowered significantly compared to $k_{p}$ [9,10]:

(2) $$k_{p,eff}=\frac{k_{p}}{1+\frac{k_{bb}}{k_{p,tert}\left[M\right]}}\;\;\;\;\;\;\;\;\;\;\;\;\;$$

While this expression is written for homopolymerization, backbiting reduces the averaged propagation rate coefficient for copolymerization in a similar fashion. Due to its increased stability, a significant fraction of the total radicals can be MCRs, even at lower temperatures when the total number of short-chain branches (SCBs) formed by the reaction is low (<1% of total polymer units). Both the MCR fraction (which influences rate) and the extent of branching are functions of the monomer concentration and temperature [11,12]. 

In our previous work, we quantified the influence of solvent choice on MA homopolymerization by using an in situ NMR technique to follow monomer conversions for batch reactions conducted in toluene and in an ethanol/water mixture. In addition, the product molar mass distributions (MMDs) and weight-average molar masses (*M_w_*) were characterized using SEC, and the percentage of short-chain branches (%SCB, the number of branch points per 100 monomer repeat units) was measured using ^13^C NMR [13]. The faster rate of polymerization observed in the ethanol/water mixture was attributed to the increased $k_{p}$ value (due to hydrogen bonding) in the ethanol/water system relative to toluene [13]. A kinetic model that included the formation and consumption of MCRs was developed to capture the influence of solvent composition ($\alpha_{\mathrm{EtOH}}$ between 0.75 and 0.95) and initial MA content ($w_{mon,0}$ between 0.10 and 0.40) on monomer conversion profiles, polymer MMDs, and branching levels over a range of operating temperatures and initiator concentrations. A second publication extended the model to MA/t-BuAAm copolymerization, accounting for the backbiting of both monomers [14]. The influence of the solvent on chain-growth kinetics was determined in a PLP-SEC study of the copolymerization system [8], with reactivity ratios estimated by fitting the drift in the comonomer composition with overall monomer conversion during batch operation [14]. The detailed model was verified by comparing predictions to the MA/t-BuAAm copolymer MMDs, comonomer compositions, and monomer conversions measured for an extensive set of batch copolymerizations [14]. 

While batch reactors are simple to operate, it is difficult to control the monomer composition and, hence, the rate of polymerization due to the unequal consumption rates of the two monomers, resulting in a broadened copolymer composition distribution. For polymerization in water, the decrease in the monomer concentration with time also leads to an increase in $k_{p}$ with conversion [15,16]. As composition heterogeneity is not desired for most copolymer applications, semi-batch operation is a common industrial practice for controlling copolymer molecular weight, dispersity, and composition. An added benefit is that controlling the monomer inventory through feeding policy provides a means to control the rate of heat generation in the system and to minimize the potential hazard of a thermal runaway [17,18]. Typical operation involves adding monomers continuously to a mixed reactor at a fixed rate such that the reactor volume and polymer content in the vessel increase. The relative amounts of monomer in the reactor are affected by both the polymerization kinetics and the rates of monomer addition, with “starved-feed” operation (i.e., maintaining the instantaneous conversion of monomer to polymer high throughout the feeding period) often used in industry to control the polymer molar mass and copolymer composition [17].

The ability of a polymerization model to represent both batch and semi-batch operations is an important test of its generality. In a batch system, there is a higher monomer concentration throughout most of the polymerization that favors chain growth (a second-order reaction with a rate proportional to the monomer concentration) over backbiting (a first-order reaction). A direct comparison of branching levels in batch and semi-batch systems has demonstrated this for the 2-hydroxyethyl acrylate (HEA)/butyl acrylate (BA) copolymerization system in an organic solvent, with the study also finding that the influence of H-bonding on backbiting differs due to the lower amount of HEA (the H-bonding monomer) in the semi-batch system [19]. The influence of relative monomer and solvent levels should be even more important for MA/t-BuAAm copolymerized in ethanol/water, as both the $k_{p}$ (Equation (1)) and $k_{p,eff}$ (Equation (2)) values in the semi-batch system will differ significantly from those in the batch system. Thus, in this study, we compare the evolution of copolymer properties (MMDs and branching levels) produced by batch and semi-batch operations, as well as verifying that the kinetic model developed from the batch study provides a good representation of semi-batch operation.

## 2. Materials and Methods

Deuterated solvents CDCl_3_ (99% purity) and methanol-D4 (MeOD-D4, 99% purity) were obtained from Sigma-Aldrich (Oakville, ON, Canada), and acetone-D6 (99.9% purity) and acetonitrile-D3 (99.8% purity) were obtained from Cambridge Isotope (Tewksbury, MA, USA); all deuterated solvents were used as received. Fully protonated solvents tetrahydrofuran (THF, ≥99.0% purity, 250 ppm BHT inhibitor, Sigma-Aldrich), ethanol-H6 (EtOH-H6, anhydrous, Commercial Alcohols, Brampton, ON, Canada), methanol-H4 (MeOH-H4, ≥99.8% purity, Sigma-Aldrich), diethyl ether (VWR Chemicals, Mississauga, ON, Canada), hexanes (≥98.5% purity, Fisher Scientific, Ottawa, ON, Canada), and deionized water (H_2_O) were also used without purification, as well as the inhibitor hydroquinone (HQ, ≥99.9% HPLC-Grade, Fisher Scientific). The thermal initiator 2,2′-azobis(2-methylpropionitrile) (AIBN, 98% purity, Sigma-Aldrich) was recrystallized in MeOH-H4 before use for lab batch experiments. It has been demonstrated that removing the inhibitor from MA (99% purity, ≤100 ppm monomethyl ether hydroquinone (MeHQ), Sigma-Aldrich) does not influence homopolymerization conversion profiles; thus, MA was used as received throughout this work, without further purification [13]. t-BuAAm (97% purity, Sigma-Aldrich), a powder at room temperature, was used as received.

Semi-batch experiments were performed at two temperatures, 60 and 70 °C, under a nitrogen atmosphere in a 3-neck 250 mL round-bottom flask (RBF) equipped with a magnetic stir bar. The reactor was maintained at the operating temperature in a thermostatted silicone oil bath heated with an IKA C-MAG HS7 hot plate for a typical reaction time of 150 min. The experimental setup was equipped with a Graham condenser; an oil bubbler for monitoring the nitrogen gas flow; and a needle valve to control the nitrogen flowrate at 30–40 bubbles/min, as observed flowing through the oil bubbler. 

A typical semi-batch reaction had a total mass of 120 g, with a portion of the solvent precharged along with the initiator to the vessel, and the remainder of the material (monomer with some solvent required to dissolve t-BuAAm) was fed at a constant rate using a REGLO Ismatec ISM4208 peristaltic pump delivered through Pharmed BPT Tubing (1.52 ID, Cole- Parmer, Montreal PQ). All components were purged for 10 min prior to the reactor and feed content preparation. After precharging with the solvent (EtOH and water) and initiator, the reactor flask was set in a silicone oil bath to heat the contents to the desired temperature under agitation, as verified by inserting a thermometer into the vessel. The reaction was initiated by the start of the monomer feed stream to the reactor.

The pump was calibrated before each reaction to deliver the desired feed at a constant rate over 150 min, although the actual delivery time sometimes varied slightly from this target. The calibration was performed by comparing the amount of fluid delivered over a 10 min period with the calculated amount and then adjusting the pump settings. The densities of MA, EtOH, and H_2_O were taken from the literature [13], and the density of t-BuAAm dissolved in solvent was assumed to be 1 g/cm^3^.

For the batch operation, the desired monomer and solvent components were added to the reactor, along with a magnetic stir bar, and purged with nitrogen for 10 min, with the pre-weighed initiator mass added once the desired reaction temperature was reached, as verified by inserting a thermometer in the vessel. 

Samples were collected at specified time intervals throughout the reaction into vials containing the HQ inhibitor, and they were immediately chilled in an ice-cold bath. A portion of the aliquots (containing the monomer, polymer, and solvent) was diluted for an NMR analysis of the monomer conversion and instantaneous comonomer composition. The remaining aliquots were dried under an air stream at room temperature and then precipitated with a 50:50 diethyl ether:hexane solution to remove residual unreacted t-BuAAm. The precipitated polymer was then dried again before preparation for an SEC analysis of the polymer MMDs and an ^1^H NMR analysis of the copolymer composition. The analytical equipment and procedures used, the proton NMR used to determine the overall monomer conversion and instantaneous comonomer composition of the reactor aliquots diluted in CDCl_3_, and the SEC used to measure the molecular mass distributions (MMD) are explained in previous studies, including the details of SEC calibration [13,14].

As previously documented [13,14], the monomer conversion profiles and polymer MMDs obtained from the batch polymerizations conducted in the stirred reactor setup were nearly identical to those obtained using the in situ NMR technique under identical conditions. For copolymerization under semi-batch operation, the overall fraction of MA in a sample can be calculated according to Equation (3), based upon the NMR determinations of the overall monomer conversion ($X_{inst})$ and the comonomer ($f_{MA}^{}$) and copolymer ($F_{MA}$) compositions:

(3) $$f_{MA,calc}=\left(1-X_{inst}\right)f_{MA}+X_{inst}F_{MA}\;\;$$

The resulting value, $f_{MA,calc}$, reflects the molar fraction of MA contained in both the unreacted monomer and the copolymer relative to the total amount of monomer (MA and t-BuAAm), a value that should equal the fraction of MA contained in the reactor feed, $f_{MA,feed}$, if the analytical methods are reliable. For all the copolymerization semi-batch experiments, the $f_{MA,calc}$ values remained within ±0.02 of $f_{MA,feed\;}$, indicating that the NMR analyses for the copolymer system are quite precise.

## 3. Results and Discussion

The MA and t-BuAAm homo- and co-polymerization kinetic models detailed in our previous publications [13,14] were fitted to represent the experimental batch data, which included monomer conversion and comonomer composition drift profiles, MMDs, and branching levels. The batch experiments were conducted in ethanol-rich ($\alpha_{\mathrm{EtOH}}$ = 0.75–1) mixtures of EtOH/H_2_O at 50–70 °C, with various initial monomer and initiator levels. The full kinetic scheme, developed assuming terminal model kinetics and considering the formation and consumption of both MA and t-BuAAm MCRs, is detailed in Table S1, with the corresponding homopolymerization kinetic coefficients summarized in Table S2. This same model, developed from the batch studies, was used to simulate the semi-batch reaction data (branching levels, residual monomer contents, and *M_w_* values) presented in the current work. As described below, primary radical termination (PRT), which was included in the initial model development but not found to effect batch polymerization behavior [13,14], had a great influence on the simulation of semi-batch operation. 

### 3.1. Homopolymerization Batch vs. Semi-Batch

**Figure 1:**
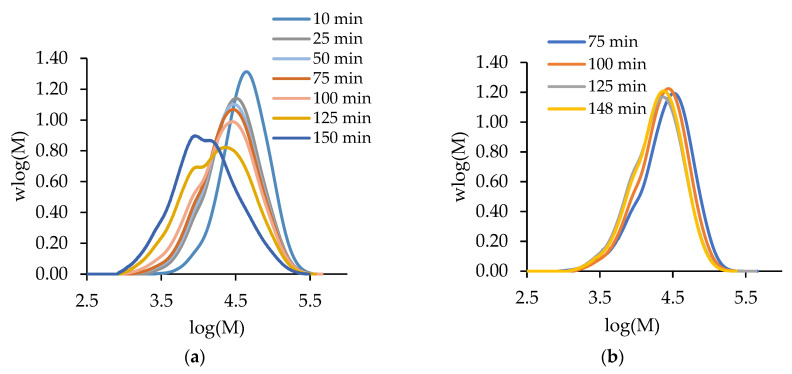
The evolution of polymer MMDs over time for (**a**) semi-batch and (**b**) batch MA homopolymerization with a final monomer/polymer content of 20 wt% synthesized in 75/25 EtOH/H_2_O at 60 °C with 1 wt% AIBN.

**Figure 2:**
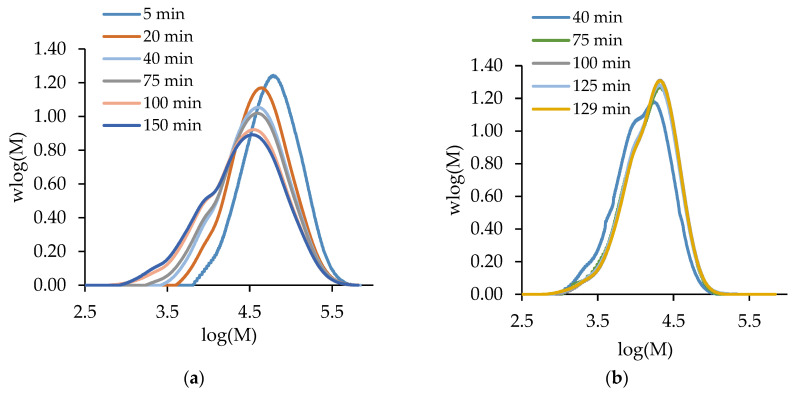
The evolution of polymer MMDs with time for (**a**) batch and (**b**) semi-batch t-BuAAm homopolymerization with a final monomer/polymer content of 10 wt% synthesized in 75/25 EtOH/H_2_O at 60 °C with 1 wt% AIBN.

Before presenting the copolymerization results, we contrast the batch and semi-batch operations for both MA and t-BuAAm homopolymerizations. Figure 1 compares the evolution of polymer MMDs with time for the batch and semi-batch MA homopolymerizations performed under identical conditions, i.e., for a final monomer/polymer content of 20 wt% in 75/25 EtOH/H_2_O at 60 °C with 1 wt% AIBN. The decrease in the monomer concentration as polymerization proceeds in a batch reactor causes both a shift to lower *M_w_* values and the broadening of the polymer MMD (Figure 1a). In contrast, the continuous feeding of the monomer throughout the reaction in semi-batch operation leads to polymer MMDs with constant positions and shapes with time (Figure 1b). Similar results are seen for tBuAAm homopolymerization batch vs. semi-batch operations, as shown in Figure 2. 

**Figure 3:**
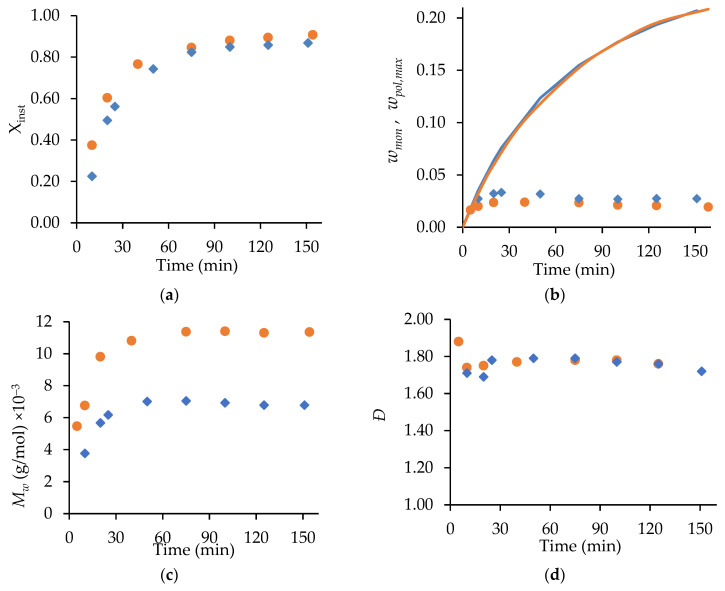
A comparison of MA (◆) and t-BuAAm (●) profiles from semi-batch homopolymerizations run under identical conditions with a final monomer/polymer content of 20 wt% in 75/25 EtOH/H_2_O with 2 wt% AIBN at 70 °C: (**a**) instantaneous conversion (*X_inst_*), (**b**) unreacted weight fraction monomer *w_mon_* (symbols) and maximum polymer content *w_pol,max_* (solid line), (**c**) *M_w_*, (**d**) Ð.

Figure 3 compares the semi-batch homopolymerizations of MA and t-BuAAm run under identical conditions. The level of unreacted monomer, $w_{mon}$, remains below 0.035 throughout for both systems, as expected for starved-feed operation; the difference between this value and the maximum polymer content, $w_{pol,max}$, in the reactor at any instant in time (Figure 3b) is a measure of the instantaneous monomer conversion (Figure 3a). However, the semi-batch homopolymerization of t-BuAAm proceeds at a faster rate than that of MA, as seen by both the higher instantaneous conversions and the lowered unreacted monomer content throughout the course of the reaction. This difference in behavior under identical reaction conditions can be understood through an examination of the rate coefficients for the two systems. The values summarized in Table 1 are calculated for 20 wt% monomer in a 75/25 EtOH/H_2_O mixture at 70 °C using the Table S2 expressions. While the value of $k_{p}$ is higher for MA than for t-BuAAm, the effective propagation rate coefficient ($k_{p,eff}$, from Equation (2)) is lowered due to the higher backbiting rate coefficient for MA. This, combined with the lower termination coefficient ($k_{t}$) for t-BuAAm, leads to the consumption of t-BuAAm being faster than that of MA, as observed in Figure 3. 

With a constant monomer feed, the $M_{w}$ profiles for both monomers remain relatively constant over time after the first 40 min (Figure 3c), with the dispersity (Ð) values for both systems constant at 1.7–1.8 (Figure 3d). The higher $k_{p,eff}$ combined with the lowered $k_{t}$ value for t-BuAAm relative to those for MA explains the higher $M_{w}$ (~11,000 g/mol) of the poly(t-BuAAm) product compared to the 6700 g/mol for poly(MA). 

**Figure 4:**
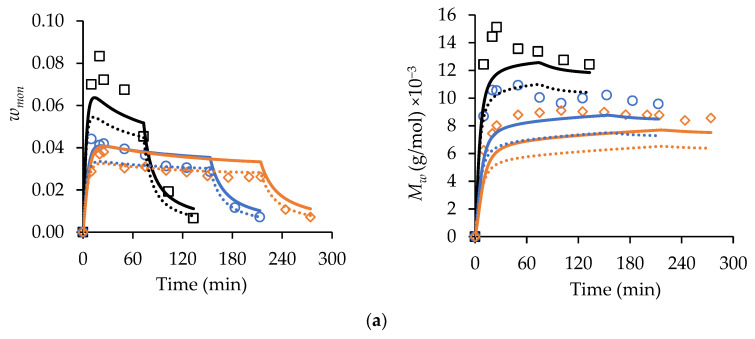

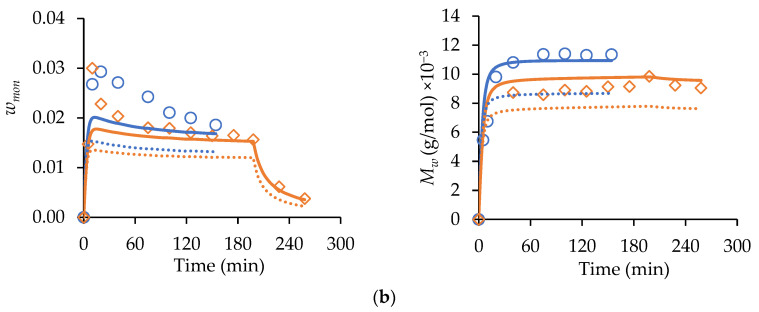
Experimental (symbols) starved-feed semi-batch *w_mon_* (left-hand column of plots) and corresponding *M_w_* (right-hand column of plots) profiles compared with simulated results with (solid lines) and without (dotted lines) primary radical termination for (**a**) MA homopolymerization with w_mon,total_ = 0.30, $\alpha_{EtOH}$ = 0.75, 2.0 wt% AIBN, 70 °C, and (**b**) t-BuAAm homopolymerization with w_mon,total_ = 0.20, $\alpha_{EtOH}\;$ = 0.75, 2.0 wt% AIBN, 70 °C. Feed times are 75 min (□), 150 min (**○**), and 225 min (**◇**). Note that the reported *w_mon,total_* and wt% AIBN are expressed relative to the total recipe in the reactor after feeding.

The ability of the kinetic scheme in Table S1 and the parameters in Table S2 to represent MA and t-BuAAm semi-batch homopolymerizations was tested by comparing predictions to measured *w_mon_* levels and polymer $M_{w}$ profiles, as shown in Figure 4. The experiments were conducted at 70 °C, with the monomer fed into the reactor at different constant rates to reach the final monomer/polymer contents of 30 wt% and 20 wt% for the MA and t-BuAAm homopolymerizations, respectively, over 75, 150, or 225 min. A constant 75/25 EtOH/H_2_O solvent composition was maintained in the feed and reactor throughout the reactions. The monomer profiles were used to estimate the rate coefficient associated with primary radical termination (PRT). This mechanism was found to affect model behavior but only under the lowered monomer concentration, which occurs with starved-feed semi-batch operation, an observation consistent with that of previous works [13,20]. While the model without PRT provided an adequate prediction of the variation in *w_mon_* for the MA homopolymerizations (with minor deviations for the experiments conducted with a 75 min feed) under semi-batch operation, the $M_{w}$ predictions were lower than the experimental values by up to 40% and 20% for the MA and t-BuAAm homopolymerizations, respectively. Furthermore, the t-BuAAm homopolymerization *w_mon_* profiles were underpredicted. Implementing PRT greatly improved the fit of $M_{w}$: 26 out of the 30 calculated values were within 25% of the experimental values for MA, and 14 out of the 17 values were within 10% of the experimental values for t-BuAAm. An improvement in the t-BuAAm *w_mon_* profiles was also observed.

The simulated conversion profiles and polymer MMs of the batch polymerizations are indistinguishable with or without the implementation of PRT, as previously noted by Agboluaje and Hutchinson [13]. During starved-feed semi-batch operation, however, the lowered reactor volume and monomer content at the start of the reaction (compared to batch) leads to a higher concentration of primary radicals, as their consumption rate via monomer addition is lowered; hence, PRT has a greater influence. The value for the PRT rate coefficient, assumed to be independent of temperature and polymer chain length, required to fit the data is an order of magnitude greater than the termination rate coefficient for two monomeric radicals $k_{t}^{MA}\left(1,1\right)$ (see Table S2) at 70 °C. As this large difference is difficult to justify based on the radical structure, there may be another (unknown) mechanism at play. Nonetheless, the implementation provides a unified representation of MA and t-BuAAm batch and semi-batch homopolymerizations and the copolymerization of the two monomers (to be discussed later). 

As found with the batch experiments [14], the branching levels could not be detected via ^13^C NMR for the t-BuAAm samples produced via the semi-batch operation; this experimental result is in agreement with the predicted final %SCB levels of <0.3% (i.e., below the ^13^C NMR detection limit). However, the SCB levels were detectable for the poly(MA) semi-batch samples collected at the end of the feed time. The experimental values were compared to simulation results in Table 2. In general, the predictions reasonably match the experimental values. As expected, increasing the feed period (which reduces *w_mon_* in the reactor) resulted in an increased level of %SCB. Furthermore, the branching levels of the polymer produced under the semi-batch operation were elevated compared to the polymer produced in the batch system; Agboluaje and Hutchinson measured a final SCB level of 3.7% for MA batch polymerization conducted with *w_mon,_*_0_ = 0.10 and *α_EtOH_* = 0.75 at 70 °C to a monomer conversion of 78% [13], compared to the ~5% SCB level measured for semi-batch experiments conducted at 70 °C under starved-feed conditions that maintain *w_mon_* < 0.04 throughout the reaction period.

### 3.2. Copolymerization Batch vs. Semi-Batch

**Figure 5:**
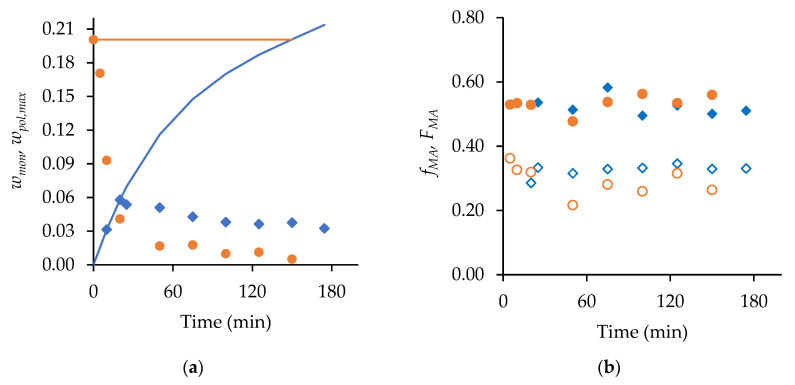
A comparison of semi-batch (◆) and batch (●) MA/t-BuAAm copolymerization with 0.50 mole fraction of MA and a final monomer/polymer content of 20 wt% in 75/25 EtOH/H_2_O with 1 wt% AIBN at 60 °C: (**a**) *w_mon_* (symbols) and *w_pol,max_* (line) vs. time, (**b**) Mole fraction of MA in copolymer (*F_MA_*, filled symbols) and unreacted comonomer mixture (*f_MA_*, open symbols) vs. time.

Batch and semi-batch operating strategies are contrasted for MA/t-BuAAm copolymerization in Figure 5. Both reactions were conducted with an equimolar ratio of the two monomers ($f_{MA,0}=0.5$ in batch and $f_{MA,feed}=0.5$ in semi-batch) in a 75/25 EtOH/H_2_O mixed solvent at 60 °C with 1 wt% AIBN. While both reactions (batch and semi-batch) had a final (monomer + polymer) content of 20 wt%, the monomer was added at a constant rate over 150 min for the semi-batch reaction. The initial reaction rate in batch was significantly faster than that in semi-batch due to the higher initial monomer concentrations. Thus, the level of unreacted monomer decreased to values lower than those of the continuously-fed semi-batch system, as seen in Figure 5a.

As shown in Figure 5b, the fraction of MA in the copolymer product ($F_{MA}≅$ 0.5) was higher than that in the unreacted monomer remaining in the reactor ($f_{MA}=$ 0.28 and 0.33 for batch and semi-batch, respectively), indicating that MA was preferentially incorporated into the polymer. The value of $f_{MA}$ decreased from its initial value of 0.50 in the batch system in the first 20 min, during which the unreacted monomer content decreased to 0.04 from its initial value of 0.20. This composition drift behavior is consistent with the reactivity ratios reported by Agboluaje et al. from in situ NMR studies as $r_{MA}$ = 1.12 and $r_{t-BuAAm}$ = 0.71 [14]. These values were found to be independent of the reaction temperature (50–70 °C), initial monomer content (10–40 wt%), and solvent composition (100/0 and 75/25 EtO/H_2_O) for the batch system, and they provided an excellent representation of the copolymer composition produced in the semi-batch system.

**Figure 6:**
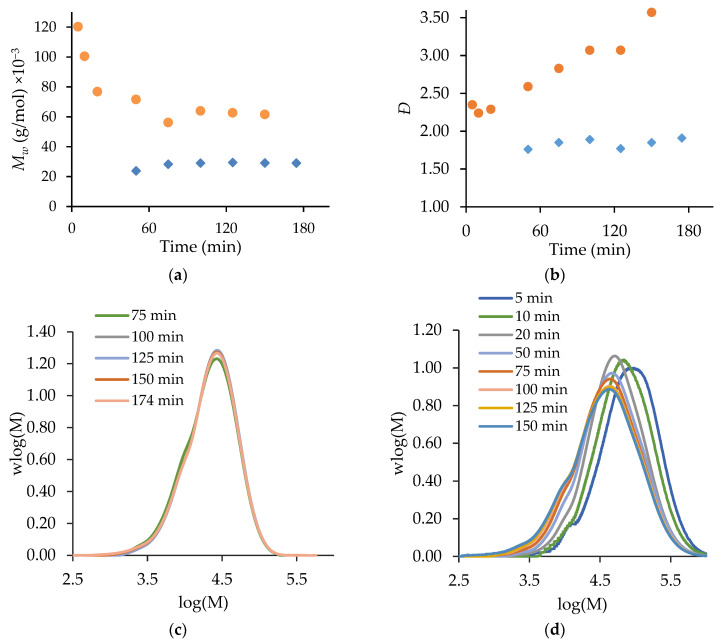
A comparison of semi-batch (◆) and batch (●) MA/t-BuAAm copolymerization with 0.50 mole fraction of MA and a final monomer/polymer content of 20 wt% in 75/25 EtOH/H_2_O with 1% AIBN at 60 °C: (**a**) *M_w_* vs. time, (**b**) Ð vs. time, (**c**) MWD vs. time for semi-batch, (**d**) MWD vs. time for batch.

The evolution of the polymer MMDs with the reaction time are contrasted for the batch and semi-batch copolymerization systems in Figure 6. There is little change in the shape or position of the semi-batch MMDs (Figure 6c), as the constant monomer level (and composition) in the reactor leads to relatively constant $M_{w}$ (24,000 to 29,000 g/mol in Figure 6a) and Ð (1.76 to 1.91 in Figure 6b) profiles. However, the MMDs of the copolymer produced in the batch reactor broaden and shift to lower values with time (Figure 6d). Thus, there is a significant decrease in polymer $M_{w}$ over time, from 120,000 to 56,000 g/mol (Figure 6a), with the values for the batch system being higher than those for the semi-batch system due to the higher monomer concentration in the early stages of the reaction. The formation of different polymer chain lengths over time also increases Ð from 2.24 to 3.57 in the batch system (Figure 6b). The capability to produce constant polymer molar masses in addition to a constant copolymer composition is a significant advantage of the semi-batch system.

**Figure 7:**
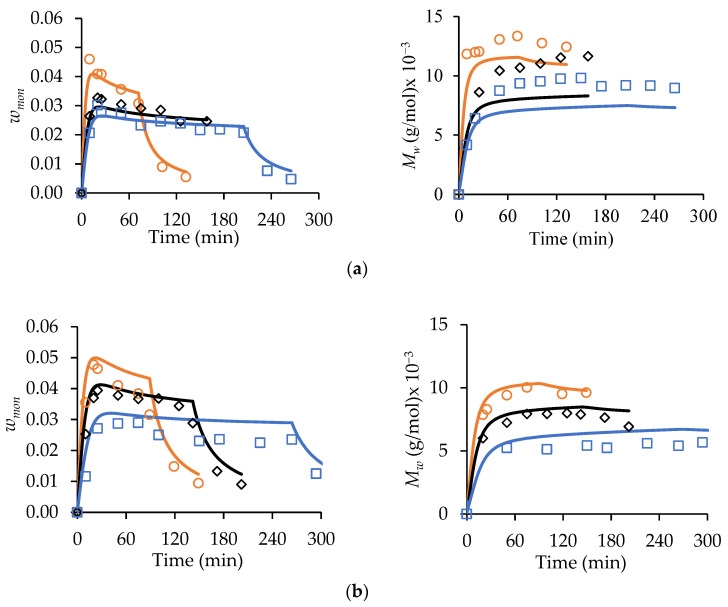
A comparison of experimental (symbols) and simulated (lines) profiles of *w_mon_* (left-hand column of plots) and polymer *M_w_* (right-hand plots) from MA/t-BuAAm starved-feed semi-batch copolymerizations conducted at 70 °C, with *w_mon,tot_* = 0.20, 2.0 wt% AIBN, and *f_MA,_*_0_ = 0.50: (**a**) *α_EtOH_* = 0.75 and (**b**) *α_EtOH_* = 1. The feed times used are 75 min (**○**), 150 min (◇), and 225 min (□). Solid lines are simulated results from PREDICI^®^ using parameters in Tables S2 and S3.

**Figure 8:**
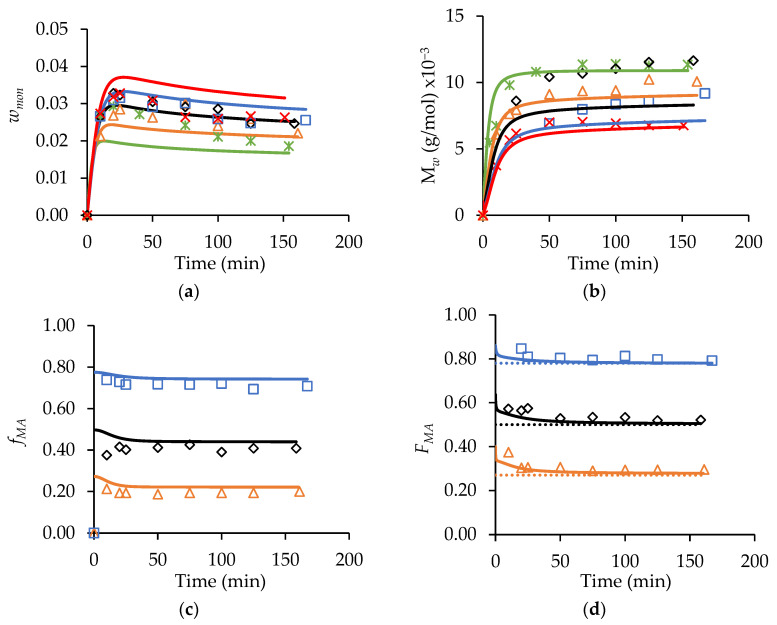
A comparison of experimental (symbols) and simulated (lines) profiles for (**a**) *w_mon_*, (**b**) polymer *M_w_*, (**c**) *f_MA_*_,_ and (**d**) *F_MA_* from MA/t-BuAAm starved-feed semi-batch copolymerizations conducted at 70 °C, with *w_mon,total_* = 0.20, *α_EtOH_* = 0.75, 2.0 wt% AIBN, fed for 150 min with varying comonomer compositions in the feed: *f_MA,_*_0_ = 0 (∗), *f_MA_*_,0_ = 0.27 (**△**), *f_MA_*_,0_ = 0.50, (◇), *f_MA,_*_0_ = 0.78 (□), *f_MA,_*_0_ = 1 (**×**). Simulations were conducted in PREDICI^®^ using the copolymerization model described in [14]; see also Table S2 and Table S3. Dotted lines in the *F_MA_* profile correspond to the feed composition (i.e., *f_MA,_*_0_) of the starved-feed semi-batch reactions.

The MA/t-BuAAm copolymerization semi-batch experiments conducted in this current work were simulated using a set of copolymerization mechanisms (Table S1) and the homopolymerization (Table S2) and copolymerization (Table S3) parameters detailed in Agboluaje et al. [14] in order to test the predictive powers of the model. The sets of experiments simulated in Figure 7 were run with different monomer feed times (75 min, 150 min, and 225 min) at 70 °C with *w_mon,tot_* = 0.20, $f_{\mathrm{MA},0}$ = 0.50, and 2.0 wt% AIBN in both ethanol/water ($\alpha_{\mathrm{EtOH}}$ = 0.75) and in pure ethanol ($\alpha_{\mathrm{EtOH}}$ = 1). Note that these *w_mon,tot_* and wt% AIBN levels are expressed relative to the total reactor contents after feeding, with a one-hour batch hold used at the end of the feeding period in order to reduce the residual monomer concentration. The experiments simulated in Figure 8 were performed using a constant feed period of 150 min at 70 °C while varying the comonomer composition of the feed ($f_{\mathrm{MA},0}$) for *w_mon,tot_* = 0.20 and 2.0 wt% AIBN in ethanol/water (*α_EtOH_* = 0.75).

In Figure 7, the *w_mon_* and $M_{w}$ values decrease as the feed time increases for both the $\alpha_{\mathrm{EtOH}}$ = 0.75 and 1 systems, as expected. A correlation between the monomer concentrations and the polymer MWs is also observed for the MA and t-BuAAm batch homopolymerizations [13,14]; the lowered monomer content increases backbiting, thus reducing both the rate of polymerization and *M_w_*. It is also seen that the w_mon_ levels are higher and that the polymer $M_{w}$ values are lowered for $\alpha_{\mathrm{EtOH}}$ = 1 (Figure 7b) compared to the *α_EtOH_* = 0.75 system (Figure 7a), as the higher $k_{p}$ values with $\alpha_{\mathrm{EtOH}}$ = 0.75 lead to an increased rate of monomer consumption. The model provides a good representation of the semi-batch *w_mon_* levels, despite the free monomer levels being substantially lower (i.e., *w_mon_* < 0.05) than those in the batch operation (*w_mon_*_,0_ = 0.10−0.40). The sharp decrease in the *w_mon_* levels observed during the 1 h batch hold period is also well-captured by the model, further validating its ability to represent the system for both batch and semi-batch operations. The $M_{w}$ profiles simulated for the 75 min feed time in Figure 7a are within 10% of the experimental values, with the model slightly underpredicting (with an average deviation of 20%) the values for the experiments conducted with the longer feed times. The model also predicts the $M_{w}$ values well for $\alpha_{\mathrm{EtOH}}$ = 1 (Figure 7b), with an average deviation of 10%.

The *w_mon_* profiles obtained using a constant feed time with varying $f_{\mathrm{MA},0}$ are also adequately represented by the model (Figure 8a). The general trends predicted for these semi-batch runs are the same as those described for the batch system: i.e., the level of unreacted monomer in the system increases as the fraction of MA in the feed is increased, an indication of a reduced reaction rate [14]. Furthermore, the $M_{w}$ values are predicted to decrease as the MA content in the copolymer increases. The experimental results follow these predictions for the most part. One anomalous result is the unexpectedly high copolymer $M_{w}$ values for the reaction conducted with $f_{\mathrm{MA},0}$ = 0.50 relative to the other profiles shown in Figure 8b, a result not matched by the model. However, as a whole, the influence of the varying comonomer composition on the copolymer $M_{w}$ values are reasonably represented, on average, within 15% of the predicted values (with the greatest deviation being observed at $f_{\mathrm{MA},0}$ = 0.50); this agreement is comparable to that observed for the modeling of the MA and t-BuAAm homopolymer semi-batch system under the same reaction conditions. Furthermore, as shown in Figure 8c and d, the model provides a good representation of both the comonomer ($f_{\mathrm{MA}}$) and copolymer ($F_{\mathrm{MA}}^{}$) composition drifts in the semi-batch system throughout the course of the reaction. $F_{\mathrm{MA}}^{}$ is maintained at a constant level close to that of the feed composition ($f_{\mathrm{MA},0}$), indicating that starved-feed operation is achieved.

## 4. Conclusions

In this study, a small-scale mixed lab reactor was used, and associated analytical techniques were developed to study the batch and semi-batch solution radical homo- and co-polymerizations of MA and t-BuAAm in EtOH and EtOH/H_2_O solvent mixtures. Polymer MMDs broadened and shifted to lower values over the course of the batch reactions as monomer concentrations decreased. In contrast, for the semi-batch operation with the monomer fed at a constant flowrate, the monomer concentration and composition remained relatively constant over the course of the reaction such that the polymer properties ($M_{w}$ and *Ð,* as well as copolymer composition) did not drift with the reaction time. Thus, this study demonstrates the advantages of the semi-batch reaction mode used widely in industry.

For the semi-batch copolymerization, the fraction of MA in the formed copolymer was higher than that in the unreacted comonomer, indicating that MA was preferentially incorporated. The polymerization rates and polymer molar masses both increased as the t-BuAAm fraction increased, due to the lowered influence of backbiting. All trends were well-captured by the model developed to represent the copolymerization system, including the influence of the EtOH/H_2_O solvent level and composition on the monomer propagation rate coefficients. Previously only tested against batch experimental data, the ability of the model to also represent semi-batch (co)polymerizations conducted at low free-monomer levels that increase $k_{p}$ values but reduce $k_{p,\mathrm{eff}}$ validates the assumptions made and the set of rate coefficients developed to describe the MA/t-BuAAm system.

## Figures and Tables

**Table 1:** Rate coefficients for MA and t-BuAAm radical homopolymerizations with 20 wt% monomer in 75/25 EtOH/H_2_O at 70 °C, as calculated from the rate expressions reported in Table S2.

Rate Coefficient	MA	t-BuAAm
$k_{p}$$\;({L\;\mathrm{mol}}^{-1}s^{-1}$)	5.1 × 10^4^	2.8 × 10^4^
$k_{bb\;}\left(s^{-1}\right)$	1055	8
$k_{p,\;tert}\;\left({L\;\mathrm{mol}}^{-1}s^{-1}\right)$	53.2	7.1
$k_{p,eff}$$\;({L\;\mathrm{mol}}^{-1}s^{-1}$)	4.5 × 10^3^	15.4 × 10^3^
$k_{t}$$\;({L\;\mathrm{mol}}^{-1}s^{-1}$)	5.5 × 10^8^	9.0 × 10^7^

**Table 2:** A comparison of measured and simulated %SCB levels from starved-feed semi-batch MA homopolymerization at 70 °C and *α_EtOH_* = 0.75. The reported *w_mon,tot_* and wt% AIBN are expressed relative to the total recipe after feeding, with the reported %SCB values for samples extracted from the reactor at the end of the feed period.

Feed Time (min)	*W_mon,tot_*	wt% AIBN	T (°C)	Experimental SCB (%)	Simulated SCB (%)
75	0.30	2.0	70	4.0	3.8
150	0.30	2.0	70	5.1	4.9
250	0.30	2.0	70	5.3	5.3

## Data Availability

The research data is summarized in this paper. Data tables will not be supplied.

## Supplementary Materials

The following supporting information can be downloaded at https://www.mdpi.com/article/10.3390/polym15010215/s1, Table S1: Full mechanism for MA/t-BuAAm copolymerization in ethanol/water solution; Table S2: Rate coefficients for modeling of MA and t-BuAAm homopolymerizations in ethanol/water; Table S3: Rate coefficients and assumptions for modeling of cross reactions implemented to represent MA/t-BuAAm copolymerizations in ethanol/water.

## References

[1] Nesvadba P., Chatgilialoglu C., Studer A. (2012). Radical Polymerization in Industry. Encyclopedia of Radicals in Chemistry, Biology and Materials.

[2] Hoessel P., Schade C., Tomlinson A., Haake H.-M., Prinz B., Schröder B. (2014). A New Polymer Generation Offering Truly Multifunctional Performance. Househ. Pers. Care Today.

[3] Jamard M., Hoare T., Sheardown H. (2016). Nanogels of methylcellulose hydrophobized with N-tert-butylacrylamide for ocular drug delivery. Drug Deliv. Transl. Res..

[4] Ganachaud F., Monteiro M.J., Gilbert R.G. (2000). Pulsed-laser Polymerization (PLP) of N- isopropyl Acrylamide (NIPAM) in Water: A qualitative study. Macromol. Symp..

[5] Agboluaje M., Refai I., Manston H.M., Hutchinson R.A., Dušička E., Urbanová A., Laćik I. (2020). A Comparison of the Solution Radical Propagation Kinetics of Partially Water-Miscible Non-Functional Acrylates to Acrylic Acid. Polym. Chem..

[6] Barner-Kowollik C., Beuermann S., Buback M., Castignolles P., Charleux B., Coote M.L., Hutchinson R.A., Junkers T., Lacík I., Russell G.T. (2014). Critically evaluated rate coefficients in radical polymerization—7. Secondary-radical propagation rate coefficients for methyl acrylate in the bulk. Polym. Chem..

[7] Vašková V., Oremusová D., Bartoñ J. (1988). Contribution to the study of acrylamide polymerization, 2. Polymerization in water/aliphatic alcohol mixtures. Makromol. Chem..

[8] Refai I., Agboluaje M., Hutchinson R.A. (2022). Radical Copolymerization Kinetics of N-tert- Butyl Acrylamide and Methyl Acrylate in Polar Media. Polym. Chem..

[9] Plessis C., Arzamendi G., Alberdi J.M., Agnely M., Leiza J.R., Asua J.M. (2001). Intramolecular Chain Transfer to Polymer in the Emulsion Polymerization of 2-Ethylhexyl Acrylate. Macromolecules.

[10] Nikitin A.N., Castignolles P., Charleux B., Vairon J.-P. (2003). Determination of Propagation Rate Coefficient of Acrylates by Pulsed-Laser Polymerization in the Presence of Intramolecular Chain transfer to Polymer. Macromol. Rapid Commun..

[11] Hutchinson R.A., Meyer T., Keurentjes J. (2005). Free-Radical Polymerization: Homogeneous. Handbook of Polymer Reaction Engineering.

[12] Asua J.M., Beuermann S., Buback M., Castignolles P., Charleux B., Gilbert R.G., Hutchinson R.A., Leiza J.R., Nikitin A.N., Vairon J.-P. (2004). Critically evaluated rate coefficients for free-radical polymerization, 5: Propagation rate coefficient for butyl acrylate. Macromol. Chem. Phys..

[13] Agboluaje M., Hutchinson R.A. (2022). Measurement and modeling of methyl acrylate radical polymerization kinetics in polar and nonpolar solvents. Ind. Eng. Chem. Res..

[14] Agboluaje M., Kaur G., Hutchinson R.A. (2022). Measurement and Modeling of N-tert -butyl Acrylamide Radical Homo- and Copolymerization with Methyl Acrylate in Ethanol/Water. Macromol. React. Eng..

[15] Preusser C., Chovancová A., Lacík I., Hutchinson R.A. (2016). Modeling the Radical Batch Homopolymerization of Acrylamide in Aqueous Solution. Macromol. React. Eng..

[16] Buback M. (2009). Propagation Kinetics in Radical Polymerization Studied via Pulsed Laser Techniques. Macromol. Symp..

[17] Wang W., Hutchinson R.A. (2008). Recent Advances in the Study of High Temperature Free Radical Acrylic Solution Copolymerization. Macromol. React. Eng..

[18] Santanakrishnan S., Tang L., Hutchinson R.A., Stach M., Lacík I., Schrooten J., Hesse P., Buback M. (2010). Kinetics and Modeling of Batch and Semibatch Aqueous-Phase NVP Free-Radical Polymerization. Macromol. React. Eng..

[19] Schier J.E.S., Cohen-Sacal D., Larsen O.R., Hutchinson R.A. (2017). The Effect of Hydrogen Bonding on Radical Semi-Batch Copolymerization of Butyl Acrylate and 2-Hydroxethyl Acrylate. Polymers.

[20] Deb P.C., Gaba I.D. (1978). Non-ideality in Vinyl Polymerisation, 2. Free-radical Polymerisation of Styrene with Azoisobutyronitrile. Makromol. Chem..

