# Supplementary material for: Measurement and Modeling of Semi-Batch Solution Radical Copolymerization of N-tert-Butyl Acrylamide with Methyl Acrylate in Ethanol/Water
Source: Polymers (Basel). 2022 Dec 31;15(1):215. doi: 10.3390/polym15010215 (PMC9824515; doi:10.3390/polym15010215)
Supplement: Supplementary file 1 [file polymers-15-00215-s001.zip › polymers-2100684-supplementary.pdf]

Supporting Information

Measurement and Modeling of Semi-Batch Solution Radical Copolymerization of *N-tert*-Butyl Acrylamide with Methyl Acrylate in Ethanol/Water

Gagandeep Kaur, Maryam Agboluaje and Robin A. Hutchinson *

Department of Chemical Engineering, Queen's University, 19 Division St., Kingston ON K7L 3N6, Canada
* Correspondence: robin.hutchinson@queensu.ca

Model Development

The full kinetic scheme, developed assuming terminal model kinetics and considering the formation and consumption of both MA and t-BuAAm MCRs, is detailed in Table S1 with the corresponding kinetic coefficients summarized in Table S2 and S3. Initiator I decomposes into two primary radicals I^* with the rate of initiator decomposition described by k_d, the rate coefficient for initiator decomposition. Radicals I^* add to M_i, monomer of species i, to form unit radical P_1^i,: $k_{p,i}^I$, the rate coefficient of monomer addition to I^* is assumed to be equal to $k_{p,i}$, the homopropagation rate coefficient of monomer i. Not all initiator radical formed will initiate new radical chains, thus this behaviour is quantified by f, the initiator efficiency. Unit radical P_1^i with M_i to form secondary radical (SPR) P_n^i, where n is the chain length. Macroradical P_n^i can either homo- or cross propagate with monomer (described by $k_{p,i}$,), transfer to solvent (described by $k_{tr,i}^{sol}$, the rate coefficient of chain transfer to solvent) thus forming a dead polymer chain D_n of chain length n and unit radical P_1^i or undergo intramolecular chain transfer (i.e. backbiting, described by the backbiting coefficient, k_{bb}) with a monomer unit within the chain thus forming midchain radical (MCR) Q_n . Monomer addition to Q_n reverts it back to an SPR, with the rate described by k_p^{tert}, the rate coefficient for monomer addition to an MCR. SPRs and MCRs disappear via bimolecular termination of SPR-SPR, SPR-MCR, MCR-MCR, SPR-I* and MCR-I* termination with the rate coefficients being $k_{t,ss}$, $k_{t,st}$, $k_{t,tt}$ and k_t^I respectively. With the exception of k_t^I, all termination rate coefficients used in this model are dependent on the viscosity of the solvent viscosity and average radical chain lengths. Initiator radicals can also recombine outside the solvent cage (also described by k_t^I) though these reactions are expected to have negligible impact on the rate of polymerization.

Table S1. Full mechanism for MA/t-BuAAm copolymerization in ethanol/water solutions.

**Initiation**
$I^* + MA \xrightarrow{k_p^{I^*MA}} P_1^{MA}$<br>$I^* + t - BuAAm \xrightarrow{k_p^{I^*t-BuAAm}} P_1^{t-BuAAm}$
**Propagation**
$P_n^{MA} + MA \xrightarrow{k_p^{MA}} P_{n+1}^{MA}$<br>$P_n^{t-BuAAm} + t - BuAAm \xrightarrow{k_p^{t-BuAAm}} P_{n+1}^{t-BuAAm}$<br>$P_n^{MA} + t - BuAAm \xrightarrow{k_p^{MA\cdot t-BuAAm}} P_{n+1}^{t-BuAAm}$<br>$P_n^{t-BuAAm} + MA \xrightarrow{k_p^{t-BuAAm\cdot MA}} P_{n+1}^{MA}$
**Transfer to monomer**
$P_n^{MA} + MA \xrightarrow{k_{tr}^{MA}} D_n + P_1^{MA}$<br>$P_n^{t-BuAAm} + t - BuAAm \xrightarrow{k_{tr}^{t-BuAAm}} D_n + P_1^{t-BuAAm}$<br>$P_n^{MA} + t - BuAAm \xrightarrow{k_{tr}^{MA\cdot t-BuAAm}} D_n + P_1^{t-BuAAm}$<br>$P_n^{t-BuAAm} + MA \xrightarrow{k_{tr}^{t-BuAAm\cdot MA}} D_n + P_1^{MA}$
**Transfer to solvent**
$P_n^{MA} + \text{Solvent} \xrightarrow{C_{tr,MA}^{sol} k_p^{MA}} D_n + P_1^{MA}$<br>$P_n^{t-BuAAm} + \text{Solvent} \xrightarrow{C_{tr,t-BuAAm}^{sol} k_p^{t-BuAAm}} D_n + P_1^{t-BuAAm}$
**SPR-SPR termination**
$P_n^{MA} + P_m^{MA} \xrightarrow{(1-\alpha_{ss}^{MA})k_{t,ss}^{MA}} D_{n+m} / \xrightarrow{\alpha_{ss}^{MA} k_{t,ss}^{MA}} D_n + D_m$<br>$P_n^{t-BuAAm} + P_m^{t-BuAAm} \xrightarrow{(1-\alpha_{ss}^{t-BuAAm})k_{t,ss}^{t-BuAAm}} D_{n+m} / \xrightarrow{\alpha_{ss}^{t-BuAAm} k_{t,ss}^{t-BuAAm}} D_n + D_m$<br>$P_n^{MA} + P_m^{t-BuAAm} \xrightarrow{(1-\alpha_{ss}^{MA\cdot t-BuAAm})k_{t,ss}^{MA\cdot t-BuAAm}} D_{n+m} / \xrightarrow{\alpha_{ss}^{MA\cdot t-BuAAm} k_{t,ss}^{MA\cdot t-BuAAm}} D_n + D_m$
**MCR-MCR termination**
$Q_n^{MA} + Q_m^{MA} \xrightarrow{(1-\alpha_{tt}^{MA})k_{t,tt}^{MA}} D_{n+m} / \xrightarrow{\alpha_{tt}^{MA} k_{t,tt}^{MA}} D_n + D_m$<br>$Q_n^{t-BuAAm} + Q_m^{t-BuAAm} \xrightarrow{(1-\alpha_{tt}^{t-BuAAm})k_{t,tt}^{t-BuAAm}} D_{n+m} / \xrightarrow{\alpha_{tt}^{t-BuAAm} k_{t,tt}^{t-BuAAm}} D_n + D_m$<br>$Q_n^{MA} + Q_m^{t-BuAAm} \xrightarrow{(1-\alpha_{tt}^{MA\cdot t-BuAAm})k_{t,tt}^{MA\cdot t-BuAAm}} D_{n+m} / \xrightarrow{\alpha_{tt}^{MA\cdot t-BuAAm} k_{t,tt}^{MA\cdot t-BuAAm}} D_n + D_m$
**SPR-MCR termination**
$P_n^{MA} + Q_m^{MA} \xrightarrow{(1-\alpha_{st}^{MA})k_{t,st}^{MA}} D_{n+m} / \xrightarrow{\alpha_{st}^{MA} k_{t,st}^{MA}} D_n + D_m$<br>$P_n^{t-BuAAm} + Q_m^{t-BuAAm} \xrightarrow{(1-\alpha_{st}^{t-BuAAm})k_{t,st}^{t-BuAAm}} D_{n+m} / \xrightarrow{\alpha_{st}^{t-BuAAm} k_{t,st}^{t-BuAAm}} D_n + D_m$<br>$P_n^{MA} + Q_m^{t-BuAAm} \xrightarrow{(1-\alpha_{st}^{MA\cdot t-BuAAm})k_{t,st}^{MA\cdot t-BuAAm}} D_{n+m} / \xrightarrow{\alpha_{st}^{MA\cdot t-BuAAm} k_{t,st}^{MA\cdot t-BuAAm}} D_n + D_m$<br>$P_n^{t-BuAAm} + Q_m^{MA} \xrightarrow{(1-\alpha_{st}^{t-BuAAm\cdot MA})k_{t,st}^{t-BuAAm\cdot MA}} D_{n+m} / \xrightarrow{\alpha_{st}^{t-BuAAm\cdot MA} k_{t,st}^{t-BuAAm\cdot MA}} D_n + D_m$

Backbiting

$$P_n^{MA} \xrightarrow{k_{bb,eff}^{MA\cdot MA}} Q_n^{MA}$$

$$P_n^{MA} \xrightarrow{k_{bb,eff}^{MA\cdot t-BuAAm}} Q_n^{t-BuAAm}$$

$$P_n^{t-BuAAm} \xrightarrow{k_{bb,eff}^{t-BuAAm\cdot t-BuAAm}} Q_n^{t-BuAAm}$$

$$P_n^{t-BuAAm} \xrightarrow{k_{bb,eff}^{t-BuAAm\cdot MA}} Q_n^{MA}$$

Monomer addition to MCR

$$Q_n^{MA} + MA \xrightarrow{k_{p,tert}^{MA}} P_{n+1}^{MA}$$

$$Q_n^{t-BuAAm} + t - BuAAm \xrightarrow{k_{p,tert}^{t-BuAAm}} P_{n+1}^{t-BuAAm}$$

$$Q_n^{MA} + t - BuAAm \xrightarrow{k_{p,tert}^{MA\cdot t-BuAAm}} P_{n+1}^{t-BuAAm}$$

$$Q_n^{t-BuAAm} + MA \xrightarrow{k_{p,tert}^{t-BuAAm\cdot MA}} P_{n+1}^{MA}$$

Primary radical termination

$$I^* + P_n^{MA} \overset{k_t^I}{\rightarrow} D_n$$

$$I^* + Q_n^{MA} \overset{k_t^I}{\rightarrow} D_n$$

$$I^* + P_n^{t-BuAAm} \overset{k_t^I}{\rightarrow} D_n$$

$$I^* + Q_n^{t-BuAAm} \overset{k_t^I}{\rightarrow} D_n$$

$$I^* + I^* \overset{k_t^I}{\rightarrow} inerts$$

Table S2. Rate coefficients for modeling of MA and t-BuAAm homopolymerizations in ethanol/water solvent. Note: w_{MA} and $w_{t\text{-}BAAm}$ are the weight fraction of MA and t-BuAAm in the solution respectively.

	**Rate Coefficient**	**Reference**
**Efficiency**	$f_{AIBN} = f_{max}\frac{[M]}{[M]+a}$, $f_{max} = 1240\exp\left(-\frac{2526}{T/K}\right)$, $a^{MA} = 1.78\cdot 10^{-12}\exp\left(-\frac{(-8940)}{T/K}\right)$, $a^{t-BuAAm} = 1.44\alpha_{EtOH}$	[1, 2]
**Initiator decomposition of AIBN**	$k_d(\mathrm{s}^{-1}) = 5.82\cdot 10^{15}\exp\left(-\frac{15920}{T/K}\right)$	[3]
**Propagation**	$k_p^{MA}(\mathrm{L\cdot mol^{-1}\cdot s^{-1}}) = k_{p,bulk}^{MA}[A + (1-A)\exp(-C(1-w_{mon})]$ $k_{p,bulk}^{MA}(\mathrm{L\cdot mol^{-1}\cdot s^{-1}}) = 1.41\cdot 10^{7}\exp\left(-\frac{2081}{T/K}\right)$ $A = 0$ $a = [-0.0075(\alpha_{EtOH} - 0.4753)^2 + 0.0164](T - T_{ref})$ $b = -0.8398(\alpha_{EtOH} - 1.7471)^2 - 0.3644$ $C = a + b$ $k_p^{t-BuAAm}(\mathrm{L\cdot mol^{-1}\cdot s^{-1}}) = k_{p,0}^{t-BuAAm}\cdot D\cdot B$ $k_{p,0}^{t-BuAAm} = 8.68\cdot 10^{6}\exp\left(-\frac{1770}{T/K}\right)$ $D = \exp[0.463(1-\alpha_{EtOH})]$, $B = \exp(-0.753w_{tBuAAm})$	[4, 5]
**Transfer to solvent**	$C_{tr,MA}^{sol} = \frac{k_{tr,MA}^{sol}}{k_p^{MA}} = 1.98\exp\left(-\frac{2813}{T/K}\right)$ $C_{tr,t-BuAAm}^{sol} = \frac{k_{tr,t-BuAAm}^{sol}}{k_p^{t-BuAAm}} = 3.76\exp\left(-\frac{3336}{T/K}\right)$	[4, 5]
**Transfer to monomer**	$C_{tr,MA}^{mon} = \frac{k_{tr}^{MA}}{k_p^{MA}} = 0.016\exp\left(-\frac{1828}{T/K}\right)$ $C_{tr,t-BuAAm}^{mon} = \frac{k_{tr}^{t-BuAAm}}{k_p^{t-BuAAm}} = 0.00118\exp\left(-\frac{1002}{T/K}\right)$	[6, 7]
**Backbiting**	$k_{bb}^{MA}(\mathrm{s}^{-1}) = 1.2\cdot 10^{8}\exp\left(-\frac{3993}{T/K}\right)$ $k_{bb}^{t-BuAAm}(\mathrm{s}^{-1}) = 2.22\cdot 10^{8}\exp\left(-\frac{5893}{T/K}\right)$	[1, 2]
**Monomer addition to MCR**	$\frac{k_{p,tert}^{MA}}{k_p^{MA}} = 0.029\exp\left(-\frac{1143}{T/K}\right)$ $\frac{k_{p,tert}^{t-BuAAm}}{k_p^{t-BuAAm}} = 0.0155\exp\left(-\frac{1412}{T/K}\right)$	[1, 6]
**SPR-SPR termination**	$< k_{t,ss} > = k_{t,ss}(1,1)\cdot < i >^{-\alpha_s} \quad i \le i_c$ $< k_{t,ss} >= k_{t,ss}(1,1)\cdot\left(i_c^{-\alpha_s+\alpha_l}\right)\cdot < i >^{-\alpha_l} \quad i > i_c$ $k_{t,ss}(1,1)(\mathrm{L\cdot mol^{-1}\cdot s^{-1}}) = A\cdot\exp\left(\frac{E_A}{RT}\right)$ $E_A = E_{A,\alpha_{EtOH}=0}\cdot viscosity_{corr,1}$	[1, 2]

	$A = A_{\alpha_{EtOH}=0} \cdot viscosity_{corr,2}$<br>$E_{A,\alpha_{EtOH}=0}(\mathrm{J \cdot mol^{-1}}) = 13850$<br>$viscosity_{corr,1} = 1 + 2.74\alpha_{EtOH} - 2.78\alpha_{EtOH}^2$<br>$viscosity_{corr,2} = \exp(11.33\alpha_{EtOH} - 11.71\alpha_{EtOH}^2)$<br><br>Parameters for MA<br>$A_{\alpha_{ethanol}=0}(\mathrm{L \cdot mol^{-1} \cdot s^{-1}}) = 1.14 \cdot 10^{11}$<br>$\alpha_s = 0.74, \alpha_l = 0.15, i_c = 25$<br>$\alpha_{ss} = 0.05$<br>Parameters for t-BuAAm<br>$A_{\alpha_{EtOH}=0}(\mathrm{L \cdot mol^{-1} \cdot s^{-1}}) = 1.89 \cdot 10^{10}$<br>$\alpha_s = 0.52, \alpha_l = 0.16, i_c = 33$<br>$\alpha_{ss} = 0.05$<br><br>These parameters are used to determine $<k_{t,ss}>$, using the CLD termination equation where $<i>$ is the average chain length of the radical.	
**MCR-SPR termination**	Parameters for MA<br>$\frac{< k_{t,st} >}{< k_{t,ss} >} = 0.58$<br>$\alpha_{st} = 0.40$<br>Parameters for t-BuAAm<br>$\frac{< k_{t,st} >}{< k_{t,ss} >} = 0.25$<br>$\alpha_{st} = 0.40$	[8, 9]
**MCR-MCR termination**	Parameters for MA<br>$\frac{< k_{t,tt} >}{< k_{t,ss} >} = 0.01$<br>$\alpha_{tt} = 1$<br>Parameters for t-BuAAm<br>$\frac{< k_{t,tt} >}{< k_{t,ss} >} = 0.01$<br>$\alpha_{tt} = 0.90$	[6, 8, 9]
**Primary radical termination**	$k_t^I(\mathrm{L \cdot mol^{-1} \cdot s^{-1}}) = 10^{10}$	[2]

Table S3. Rate coefficients and assumptions for modeling of cross reactions implemented to represent MA/t-BuAAm copolymerization in EtOH/H2O with α_{EtOH} = 0.75 and 1.

	**Rate Coefficient**	**Reference**
**Efficiency**	$f_{AIBN,\,average} = f_{MA} f_{AIBN}^{MA} + f_{t-BuAAm} f_{AIBN}^{t-BuAAm}$	This work.
**SPR propagation**	$k_p^{MA\cdot t-BuAAm}(\mathrm{L\cdot mol^{-1}\cdot s^{-1}}) = \frac{k_p^{MA}}{r_{MA}}$<br>$k_p^{t-B\qquad \cdot MA}(\mathrm{L\cdot mol^{-1}\cdot s^{-1}}) = \frac{k_p^{t-BuAAm}}{r_{t-BuAAm}}$<br>$r_{MA}$=1.12, $r_{t-BuAAm}$= 0.71	This work.
**Cross transfer to monomer**	$k_{tr}^{ij}(\mathrm{L\cdot mol^{-1}\cdot s^{-1}}) = \left(\frac{k_{tr}^{jj}}{k_p^{jj}}\right) k_p^{ij} = C_{tr}^{j}\frac{k_p^{ii}}{r_{ji}}$	[10, 11]
**Cross backbiting**	$k_{bb,\,eff}^{ij}(\mathrm{s^{-1}}) = F_j^{inst}\cdot k_{bb}^{ij}$<br>Where $k_{bb}^{ij}(\mathrm{s^{-1}}) = k_{bb}^{j}\frac{k_p^{ii}}{r_{ji}k_p^{jj}}$<br>For $k_{bb,\,eff}^{i\cdot t-B}$ where *i*=MA or t-BuAAm<br>$k_{bb,\,eff}^{ij}(\mathrm{s^{-1}}) = F_j^{inst}\cdot k_{bb}^{ij}$<br>For $k_{bb,\,eff}^{t-BuAAm\cdot j}$ where *j*=MA<br>$k_{bb,eff}^{ij}(\mathrm{s^{-1}}) = 0.10\cdot F_j^{inst}\cdot k_{bb}^{ij}$<br>For $k_{bb,eff}^{ij}$ where *i*=*j*=MA<br>$k_{bb,eff}^{ij}\ (\mathrm{s^{-1}}) = (1 - F_{t-BuAAm}^{inst})\cdot F_j^{inst}\cdot k_{bb}^{j}$	This work, [10, 11]
**Cross monomer addition to MCR**	$k_{p,tert}^{ij}(\mathrm{L\cdot mol^{-1}\cdot s^{-1}}) = k_p^{ij}\frac{k_{p,tert}^{jj}}{k_p^{jj}} = k_p^{ii}\frac{k_{p,tert}^{jj}}{r_{ji}k_p^{jj}}$	[10, 11]
**Cross termination**	$< k_{t,xx}^{ij} > (\mathrm{L\cdot mol^{-1}\cdot s^{-1}}) = \left(< k_{t,xx}^{i} > \cdot < k_{t,xx}^{j} >\right)^{1/2}$<br>Where *xx*= *ss*, *st* or *tt* (ie. radical types that participate in termination)<br>$\alpha_{ss}^{MA\cdot t-BuAAm} = 0.05$<br>$\alpha_{st}^{MA\cdot t-BuAAm} = \alpha_{st}^{t-BuAAm\cdot MA} = 0.40$<br>$\alpha_{tt}^{MA\cdot t-BuAAm} = 0.90$	[10, 11]

References

[1] M. Agboluaje and R. A. Hutchinson, "Measurement and modeling of methyl acrylate radical polymerization kinetics in polar and nonpolar solvents," *Ind. Eng. Chem. Res.,* 2022.

[2] M. Agboluaje, G. Kaur and R. A. Hutchinson, "Measurement and Modeling of N-tert -butyl Acrylamide Radical Homo- and Copolymerization with Methyl Acrylate in Ethanol / Water," *Macromol. React. Eng.,* vol. in. press., pp. 1-16, 2022.

[3] "AIBN," FUJIFILM Wako Pure Chemical Corporation, [Online]. Available: https://specchem-wako-jp.fujifilm.com/en/oilazo/AIBN.htm. [Accessed September 2021].

[4] M. Agboluaje, I. Refai, H. H. Manston, R. A. Hutchinson, E. Dušička, A. Urbanová and I. Lacík, "A comparison of the solution radical propagation kinetics of partially water-miscible non-functional acrylates to acrylic acid," *Polym. Chem.,* vol. 11, no. 44, pp. 7104-7114, 2020.

[5] I. Refai, M. Agboluaje and R. A. Hutchinson, "Radical Copolymerization Kinetics of N-tert-Butyl Acrylamide and Methyl acrylate in Polar Media," *Polym. Chem.,* vol. 13, no. 14, pp. 2036-2047, 2022.

[6] C. Preusser, A. Chovancová, I. Lacík and R. A. Hutchinson, "Modeling the Radical Batch Homopolymerization of Acrylamide in Aqueous Solution," *Macromol. React. Eng.,* vol. 10, no. 5, pp. 490-501, 2016.

[7] S. Maeder and R. G. Gilbert, "Measurement of Transfer Constant for Butyl Acrylate Free-Radical Polymerization," *Macromolecules,* vol. 31, no. 14, pp. 4410-4418, 1998.

[8] N. F. Wittenberg, C. Preusser, H. Kattner, M. Stach, I. Lacík, R. A. Hutchinson and M. Buback, "Modeling Acrylic Acid Radical Polymerization in Aqueous Solution," *Macromol. React. Eng.,* vol. 10, no. 2, pp. 95-107, 2016.

[9] H. Kattner and M. Buback, "Termination, Propagation, and Transfer Kinetics of Midchain Radicals in Methyl Acrylate and Dodecyl Acrylate Homopolymerization," *Macromolecules,* vol. 51, no. 1, pp. 25-33, 2018.

[10] I. H. Ezenwajiaku, Zigelstein, Rachel, Chovancová, Anna, Lacík, Igor and Hutchinson, Robin A., "Experimental and Modeling Investigations of Aqueous-Phase Radical Copolymerization of 2-(Methacryloyloxyethyl)trimethylammonium Chloride with Acrylic Acid," *Ind. Eng. Chem. Res.,* vol. 59, no. 8, pp. 3359-3374, 2020.

[11] C. Preusser, "Kinetics and Modeling of Free Radical Aqueous Phase Polymerization of Acrylamide with Acrylic Acid at varying Degrees of Ionization," Queen's University, Kingston, 2015.
